# Assessing the effects of native and alien plant ash on mosquito abundance

**DOI:** 10.1002/ece3.9371

**Published:** 2022-10-01

**Authors:** Vincent T. Netshituni, Ross N. Cuthbert, Farai Dondofema, Tatenda Dalu

**Affiliations:** ^1^ Aquatic Systems Research Group, Department of Geography and Environmental Sciences University of Venda Thohoyandou South Africa; ^2^ School of Biological Sciences Queen's University Belfast Belfast UK; ^3^ South African Institute for Aquatic Biodiversity Makhanda South Africa; ^4^ Aquatic Systems Research Group, School of Biology and Environmental Sciences University of Mpumalanga Nelspruit South Africa; ^5^ Wissenschaftskolleg zu Berlin ‐ Institute for Advanced Study Berlin Germany

**Keywords:** aquatic ecosystem, colonization, invasion, macroinvertebrates, wildfire

## Abstract

Plant invasions have been linked to displacement of native vegetation and altering of fire regimes and might influence vector mosquito populations by altering habitats or nutrient inputs. Whereas wildfire effects on terrestrial ecosystems are relatively well‐studied, ash depositions into aquatic ecosystems and effects on semi‐aquatic taxa such as mosquitoes have remained overlooked. Here, we investigated mosquito colonization in water treated with ash from native plants [quinine tree (*Rauvolfia caffra*), Transvaal milk plum (*Englerophytum magalismontanum*), apple leaf (*Philenoptera violacea*)] and invasive alien plants [i.e., lantana (*Lantana camara*), guava (*Psidium guajava*), red river gum (*Eucalyptus camaldulensis*)] in containers at two ash concentrations (i.e., 1, 2 g/L)*.* Overall, there was no statistically clear difference in colonization between ash from native and alien species. We recorded colonization by two mosquito genera (*Culex* spp. and *Anopheles* spp.), with *Culex* generally much more abundant than *Anopheles*. Few differences were identified among the plants, with statistically clear effects of ash type and concentration on larval and pupal stages. High *Culex* egg and larval abundances were shown in lantana and apple leaf treatments compared to controls, and milkplum versus controls for pupae of both genera. Further research is required to elucidate the influence of nutrient inputs from different ash species on vector mosquito population dynamics.

## Graphical abstract

Increasing wildfires could alter aquatic communities via ash inputs. We examined the influence of native and alien plant ash on mosquito abundance, finding signficiant effects on ovipostion.

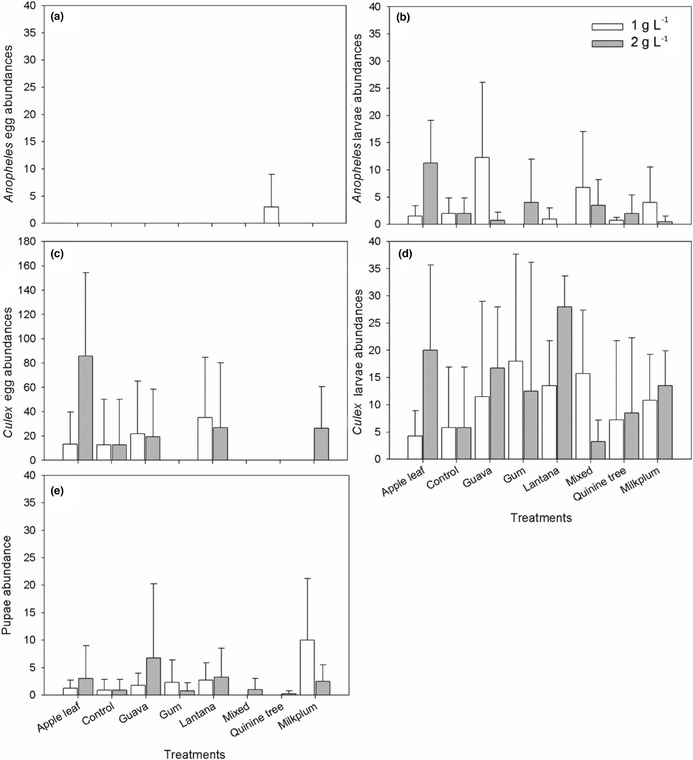

## 1 INTRODUCTION

Wildfires have attracted increasing attention as climates change, particularly due to their direct and indirect impacts on ecosystems. Invasive alien plants have been responsible for altering fire regimes in many biomes (Brooks et al., 2004). Plant invasions are a growing concern across geographic regions and habitat types, causing both negative and positive ecological and economic impacts (Vilà et al., 2011). There are numerous negative impacts relating to these invaders, including excessive utilization of water (Calder and Dye, 2001), alteration of water quality (Chamier et al., 2012), and restructuring and displacement of native species (Hejda et al., 2011). Invasive alien plants have been, in some cases, linked with human health implications achieved through two mechanisms, i.e., production of biotoxins/allergens and provision of suitable habitat for pathogen/parasite vectors (Mack et al., 2000; Mazza et al., 2014). Invasive alien plants such as lantana (*Lantana camara*) tend to increase the frequency of wildfires by providing greater biomass that is easier to ignite than native species (Bell et al., 2009; Berry et al., 2011).

In South Africa alone, over 200 alien plants are considered invasive, with most of them occurring within riparian environments (Chamier et al., 2012). Hence, this leaves the riparian vegetation, together with its helophyte communities, exposed to invader‐related impacts such as wildfires (Pettit & Naiman, 2007; Pinto et al., 2004). Globally, wildfires have gained much attention as one of the sources of contaminants to aquatic ecosystems due to their associated production and deposition of foreign substances, i.e., ash, into adjacent aquatic environments (Silva et al., 2015). Water quality in aquatic ecosystems usually decreases following wildfires (Kinoshita et al., 2016; Kristensen et al., 2014) with significant implications for the ecosystem's functionality.

We studied three widespread invasive plants in South Africa: lantana *L. camara*, gum *Eucalyptus camaldulensis*, and guava *Psidium guajava*. The records of lantana in South Africa date back to 1858 with the current infestations expected to spread over a wider range due to climate change and dispersal by birds and water (Vardien et al., 2012). About two million hectares were declared lantana‐infested in the year 2000 (Le Maitre et al., 2000). Globally, *L. camara* poses a wide range of impacts in ecosystems it infests; for instance, elevated fire frequency and intensity (Berry et al., 2011), reduced water quality (River Health Programme, 2003), high water usage (Le Maitre et al., 2016), and displacement of native vegetation. Very little is known about the red river gum *E. camaldulensis* invasion history in South Africa, but its introduction dates to 1870, and it has become the most widespread invasive eucalypt among all species in South Africa (Hirsch et al., 2020), and is well established along water courses (Forsyth et al., 2004). Eucalypts including *E. camaldulensis* are speculated to have a fire risk hazard, especially crown fires (Hirsch et al., 2020). This eucalypt also poses allelopathic effects and displaces native vegetation (Ruwanza et al., 2015), alters soil physicochemical properties (Tererai et al., 2015), and has high water usage (Le Maitre et al., 2016). However, it is also used for numerous other benefits such as timber, firewood, etc. (Forsyth et al., 2004). Similarly, *P. guajava* is a plant of many uses. Guava was introduced to South Africa by European settlers as a crop (Anthony et al., 2011). In most South African biomes, guava has been identified as the most important invasive species, particularly for its uses (Anthony et al., 2011), however, the negative environmental impacts of guava are not well documented. Guava impacts include economic loss due to removal (Anthony et al., 2011), hosting of pathogens (Mwatawala et al., 2006), allelopathic effects (Chapla & Campos, 2010), and displacements of native species.

Macroinvertebrates, among other aquatic organisms, are affected by a decrease in water quality, mainly due to the introduction of foreign substances (Xu et al., 2014), such as ash. These effects can harbor changes in species diversity, richness, and composition (Pinto et al., 2004; Rizo‐Patrón et al., 2013). After a disturbance in aquatic ecosystems, macroinvertebrates may also display preferential colonization of affected areas within the ecosystem (Beckett & Miller, 1982; Vaz et al., 2014). Moreover, the recovery of streams to pre‐fire conditions takes time (i.e., up to 10 years) depending on the stream (Verkaik et al., 2013) and may depend mainly on the time between fire and postfire flows together with their magnitudes (Verkaik et al., 2015).

Mosquitoes are external colonists to various waters (i.e., ovipositing eggs from the terrestrial realm), from small water containers to large water bodies (Caillouët et al., 2008; Medlock & Vaux, 2013), with contaminated waters being most likely to be colonized by certain mosquitoes, such as *Culex* (Ozeri et al., 2020; Vonesh & Kraus, 2009). Water is essential for the early life stages of mosquitoes (Dale & Knight, 2008); specifically, all larvae and pupae develop in water until the adult stage (Harbach & Besansky, 2014), whereas eggs can be deposited directly in water or land that will be flooded. Mosquitoes are mostly a risk and pest to humans and wildelife by disease transmission and nuisance biting (Dale & Knight, 2008; Jupp, 2005). However, they also have a significant ecological role in the environment. They are a prey item for other species in food webs (Fang, 2010) and secondarily pollinate certain plants (Harbach & Besansky, 2014).

In this study, we analyzed the differences in mosquito abundances after colonization in water treated with ash from three native (i.e., quinine tree, transvaal milk plum, and apple leaf) and three invasive alien plants (i.e., lantana, guava, and gum) plants. The plant species selected for the study usually occur near water resources in South Africa; thus, ash produced from these plants during fires is more likely to be deposited or leached into adjacent aquatic environments. The study aimed to investigate the relationship between wildfires and mosquito abundances, by assessing how ash generated from different alien and native plant species with two concentrations (i.e., 1 and 2 g/L) may influence the abundance of early stages of mosquitoes (i.e., eggs, larvae, and pupae) in waters. We hypothesize that ash will positively influence the abundance of mosquitoes owing to the reduced habitat suitability which may attract adult mosquitoes for oviposition and benefit their development. Moreover, we posit that invasive alien plants will further promote greater abundances of vector mosquitoes than the native plant species, given the provisioning of higher biomass.

## 2 MATERIALS AND METHODS

### 2.1 Experimental design

The experiment was conducted at the University of Venda Department of Geography and Environmental Sciences Atrium (−22.977550, 30.443851). The climate of the study area is classified as a humid and subtropical, with the average rainfall ranging between 400 and 800 mm and peaking between January and February. The average temperature during the warm season is just above 28°C and just below 24°C for the cold season; the temperatures ranged between 26 and 30°C during the experiment. The study was conducted using 64 × 12 L buckets (⌀ 25 cm, 30 cm depth). The buckets were placed and filled with 10 L filtered (63 μm mesh to remove zooplankton) river water collected from the Mvudi River (−22.983544, 30.443331). The point of water collection was at the riffles; the discharge of the river was approximately 5000 L/min. The river is a shallow stream, about a few meters deep from the banks. The river portion where water was collected and a few kilometers up and downstream can be classified as urban. In the region, wildfires usually occur at a smaller scale, often arising from agricultural fields or home steads, but during water collection, we did not identify any fire activities. Water was collected and stored in a quarter‐full 1000 L square container and then transported to the area of the experiment. The container was emptied into the experimental buckets. Five grams of fertilizer (3:2:1 N:P:K ratio; Wonder Garden Care, Johannesburg) were added into the water to facilitate ‘baseline’ phytoplankton growth over 30 days before the start of the experiment.

Twigs with leaves were collected from three native (i.e., *R. caffra*, *E. magalismontanum*, *P. violacea*) and three invasive alien (i.e., *L. camara*, *P. guajava*, *E. camaldulensis*) plants before being sundried for 40 days in an open yard at Thohoyandou Unit C (September to October 2020). Once the leaves and twigs had dried, each plant species was separately placed inside a metal bucket, then ignited with a matchstick and allowed to burn for 50–60 min to produce ash. The fire intensity was not standardized across the species, but adequate in each case to produce a representative ash sample for experimentation. The fire was extinguished by covering each metal bucket with a lid. All of the ash was collected separately per species after it had cooled down and then placed into labeled zip lock bags to form the six individual ash treatments (i.e., per species) and an additional seventh treatment (mixed) using equal proportions of the other six individual ash treatments.

The experiment used a randomized design, with eight species treatments [i.e., 3 native, 3 alien, 1 mixed, 1 control (no ash)] × 4 replicates × 2 ash concentrations (i.e., 1, 2 g/L), and was run from November 05, 2020 to December 10, 2020. Ash at 10 g or 20 g mass was randomly added into the individual buckets except for controls where no ash was introduced. The ash mass was chosen to assess the different mosquito abundances under varying ash concentrations. To compensate for water loss, borehole water was used to top up the buckets to initial levels. These water additions were well‐balanced among buckets.

After 6 weeks, each bucket was strained through a sieve (63 μm), and all the bucket contents were collected into small containers with 30 ml of 70% ethanol. Contents collected from buckets were placed under a microscope (Zeiss Stemi 2000‐C) and observed between 20× and 30× magnification. Mosquitoes were counted and recorded as eggs and larvae for *Culex* spp. and *Anopheles* spp. Pupae, however, were recorded in combination for both genera as they did not exhibit prominent morphological differences. All the eggs were counted individually. Mosquito genera were identified using a mosquito morphology guide (Becker et al., 2010).

### 2.2 Statistical analysis

Generalized Linear Models (Poisson log‐linear regression model) were used to analyze the count variables against two factors, i.e., treatment (six species + mixed + control) and concentration (1 g/L + 2 g/L) for main effects, and their interaction. The count variables were grouped by genera and life stage, and pupae were analyzed altogether (i.e., for both genera). Therefore, five models were fit in total (two species × two life stages + pupae). Type I sum of squares was used for deviance analysis and assessment of statistically clear levels of the main effects at *p* < .05. Estimated Marginal Means for factors and their interactions were computed following Least Significant Difference for pairwise comparison with Tukey adjustment. Counts were similarly analyzed coarsely against another factor, i.e., species type (alien + native) with other factors pooled together. All statistical analysis was done using SPSS, version 24 (IBM Corp., 2016).

## 3 RESULTS

**FIGURE 1:**
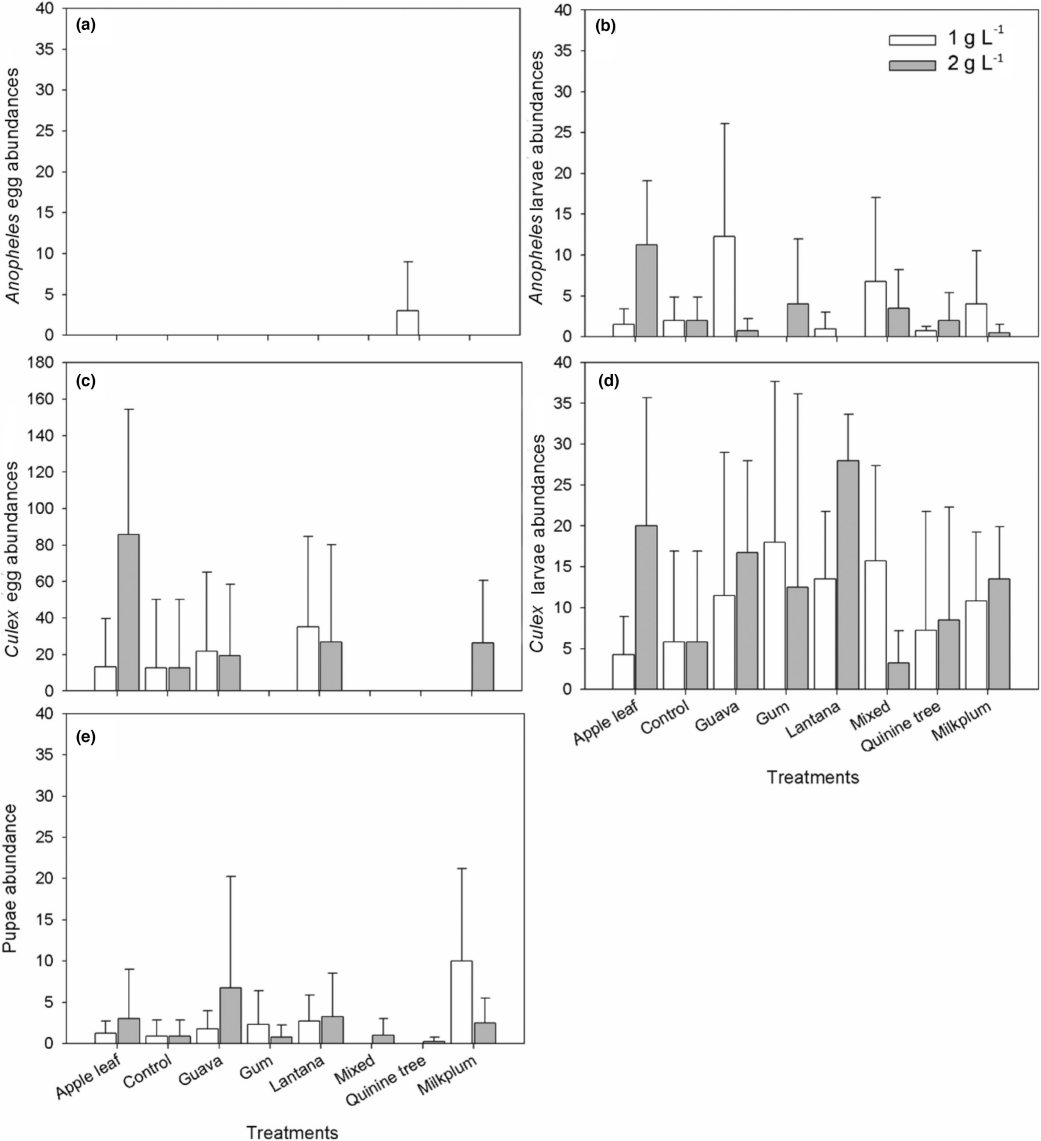
Mean (±SD) *Anopheles* spp. eggs (a) and larvae (b); *Culex* spp. eggs (c) and larvae (d), and pupae (e) abundances among the different experimental treatments. native: Quinine tree, Transvaal milk plum, apple leaf; alien: Lantana, guava, gum.

We recorded colonization by two mosquito genera (*Culex* spp. and *Anopheles* spp.), with *Culex* spp. most abundant (Table 1). An analysis between alien‐native plant groups indicated no statistically clear differences between plants when pooled according to invasion history (Table 2). *Anopheles* and *Culex* larvae differed significantly among treatments (ash types) and concentrations, while pupae only differed among treatments (Table 2). Egg differences were not statistically clear for *Culex*, or insufficient in numbers for the *Anopheles* analysis (Table 2; Figure 1).

**TABLE 1:** Overall mosquito abundances for eight level treatment; six plant species (apple leaf, guava, gum, lantana, quinine tree, and transvaal milkplum) + mixed + control under two ash concentrations (1 and 2 g/L).

	*Culex* spp.		*Anopheles* spp.		Pupae
Treatment	Egg	Larvae	Egg	Larvae	(combined)
(a) 1 g/L					
Apple leaf	53	17	0	6	5
Control	0	52	0	13	7
Guava	87	46	0	49	7
Gum	0	54	0	0	7
Lantana	141	54	0	4	11
Mixed	0	63	0	27	0
Quinine tree	0	29	12	3	0
Transvaal milkplum	0	54	0	20	50
(b) 2 g/L					
Apple leaf	343	80	0	45	12
Control	113	52	0	5	1
Guava	78	67	0	3	27
Gum	0	50	0	16	0
Lantana	107	112	0	0	14
Mixed	0	13	0	14	6
Quinine tree	0	34	0	8	1
Transvaal milkplum	105	54	0	2	10

**TABLE 2:** Main effects of the mosquito abundances from six models, with eight level treatment; six plant species (apple leaf, guava, gum, lantana, quinine tree, and transvaal milkplum) + mixed + control and two ash concentrations (1 and 2 g/L).

Model	Term	Chi‐square	df	*p*‐value
Alien‐native	Ash type	0.06	1	.81
*Anopheles* eggs	Ash type	–	–	–
	Concentration	–	–	–
	Ash type × concentration	–	–	–
	Plant type	–	–	–
*Culex* eggs	Ash type	5.99	4	.19
	Concentration	0.49	1	.49
	Ash type × concentration	2.85	2	.24
*Anopheles* larvae	Ash type	19.95	7	**.01**
	Concentration	0.26	1	.61
	Ash type × concentration	23.74	4	**.00**
*Culex* larvae	Ash type	16.73	7	**.02**
	Concentration	1.01	1	.32
	Ash type × concentration	16.52	1	**.01**
Pupae	Ash type	20.81	7	**.01**
	Concentration	0.05	1	.82
	Ash type × concentration	7.20	4	.13

Bold values represent significance (*p* < .05).

For 1 g/L *Anopheles* eggs were only recorded in quinine tree. No larval *Anopheles* were recorded in gum, and this yielded a statistically clear difference in contrast to controls (*p =* .027) and in the control vs lantana comparison (*p* = .027). High abundances of *Anopheles* were recorded in guava and low abundances in quinine trees generally (Figure 1).

At 1 g/L, no *Culex* eggs were recorded in gum, mixed, quinine tree, and milkplum ash treatments. However, high abundances were recorded in lantana, with low abundances recorded in controls. *Culex* larval mosquitoes were recorded in all treatments, with high abundances in the gum and low abundances in the apple leaf (Figure 1). There were no significant differences compared to controls.

Mixed and quinine treatments did not show the occurrence of pupal mosquitoes under 1 g/L treatments. However, milkplum recorded higher abundances in overall abundance than other treatments, while low abundances were recorded in controls (Figure 1), as a result statistical significance occurred between these two treatments (*p =* .044).

For the 2 g/L treatments, no *Anopheles* spp. eggs were recorded. However, larvae were recorded in all treatments except for lantana, with high abundances recorded in apple leaf (Figure 1) and a clear difference was observed between apple leaf vs controls (*p* = .01).

Apple leaf had the highest *Culex* egg abundances at this concentration with no eggs found in gum, mixed, or quinine groups. *Culex* larvae were recorded in all the treatments, with lantana recording high abundance and mixed ash recording low abundance (Figure 1), resulting in clear statistical significance observed between lantana and controls (*p* < .01).

Pupae were recorded in all 2 g/L treatments, with increased abundances in guava and low quantities in the quinine tree (Figure 1).

## 4 DISCUSSION

Plant invasions are considered a severe concern to ecosystems due to their numerous adverse impacts such as alteration of fire regimes globally and potential deposition of ash into aquatic environments. In the current study, we assessed how different ash types generated from alien plants compared to native plants with two concentrations (1 and 2 g/L) might influence the abundances of early stages of mosquitoes in the water. We also assessed the effects of ash concentration on their abundance after colonization.

Overall, no clear pattern was demonstrated between native and alien species' influence on the mosquito abundances, similarly, no clear pattern was demonstrated by the ash concentration choices for this study. The variations in *Culex* eggs caused by plants such as apple leaf could have been coupled by habitat suitability i.e., patch size (Bohenek et al., 2017) and preferences (Lampman & Novak, 1996), attracting more female *Culex* mosquitoes for oviposition. Alternatively, there is a high possibility that the overall abundance may have been influenced by attractants such as nutrients and food availability in the early stages, but these require further elucidation. The *Culex* mosquitoes are usually the fastest to colonize waters (Williams et al., 1993), which may grant them a chance to successfully colonize newly established or disturbed aquatic environments.

In the current study, we recorded high abundances of *Culex* mosquitoes compared to *Anopheles*, and we thus concluded that the environmental conditions i.e., temperature, and the entire water chemistry (Kinga et al., 2022) greatly attracted *Culex* mosquitoes over *Anopheles* mosquitoes. However, in the other studies conducted in Sudan (Seufi & Galal, 2010), South Africa (Munhenga et al., 2014), and Tanzania (Emidi et al., 2017), high abundances of *Anopheles* mosquitoes have been recorded during the same period (November–December), probably because they are not container breeders and possibly prefer cleaner water (Munga et al., 2005) compared to the *Culex* mosquitoes that are usually found in high abundances in waters with impurities (Medlock & Vaux, 2014). For that reason, we would have expected to record more *Anopheles* mosquitoes in controls. Nonetheless, the densities for *Anophele*s mosquitoes are known to be seasonal across regions and could relate to their oviposition of eggs singularly compared to that in batches as in *Culex*.

There were no statistically clear effects recorded among ash groups (treatments). Nonetheless, the *Culex* mosquitoes generally increase in abundance with increased ash concentration; in apple leaf, guava, and lantana. This may indicate a potential risk to public health in cases where ash is leached into water bodies in large quantities, following the evidence that *Culex* mosquitoes have been reported to be one of the primary vectors of the Rift Valley Fever virus (Seufi & Galal, 2010), however, this needs further studies with wide range of concentrations. Although not assessed in the current study, it cannot be entirely ruled out that the ash could have exhibited insecticidal effects at some level probably due to plant extracts (Kamaraj et al., 2011). Indeed, some plant extracts have been linked with insecticidal effects on vector mosquitoes (Niroumand et al., 2016; Sakthivadivel & Daniel, 2008). Moreover, such effect may vary depending on the plant part i.e., leaves, stem, or flowers (Alfaki, 2015), thus we could have observed more patterns in mosquito abundances for similar species with ash produced from different plant parts. In the three life stages considered (i.e., egg, larvae, and pupae), the pupal stage is usually the shortest; there is a possibility that the sampling period might have missed the short window before transformation into adult mosquitoes. Moreover, we had expected to record *Aedes* spp. as they are commonly found in the experimental area and are familiar colonists in container‐style habitats (Jupp, 2005). Their lack of recording was likely because they oviposit above the water line with the water level not increasing over the experiment and promoting hatching.

There is uncertainty regarding the tolerable ash concentration levels of the identified mosquitoes. Similarly, there are also uncertainties concerning the long‐term effects of ash deposited into aquatic ecosystems on the abundance of mosquitoes. However, the ecological drivers of mosquito population in colonizing successfully are likely to depend on local conditions rather than the broad regional scale, and the effect of ash on mosquito abundances may vary from plant to plant probably owing to plant ash chemical composition rather than history of occurrence and distribution.

## 5 CONCLUSION

The study assessed the effects of two ash concentrations on mosquito abundances using six plant species, three of which are native and three are invasive and alien. We recorded higher surpluses of *Culex* spp. in contrast with *Anopheles* spp., probably owing to habitat selectivity and egg‐laying behavior. The possibility of season playing a role cannot be ruled out either. Generally, the ash generated from the selected species had no notable and consistent effects on the abundance of mosquitoes. The ash concentration seemingly played a role at times in the abundance of *Culex* spp. as the abundance slightly increased with the increasing concentration, indicating a potential risk of vector mosquitoes that may in turn cause implications to human health. Nonetheless, further study is required to assess the ash elements that are likely to attract or repel mosquitoes and exploring different water sources or habitat types.

## AUTHOR CONTRIBUTIONS

**Vincent T. Netshituni:** Formal analysis (equal); investigation (equal); methodology (equal); validation (equal); visualization (equal); writing – original draft (equal). **Ross N. Cuthbert:** Conceptualization (equal); supervision (equal); writing – review and editing (equal). **Farai Dondofema:** Conceptualization (equal); project administration (equal); supervision (equal); writing – review and editing (equal). **Tatenda Dalu:** Conceptualization (equal); funding acquisition (equal); project administration (equal); supervision (equal); visualization (equal); writing – review and editing (equal).

## CONFLICT OF INTEREST

The submitted work was not carried out in the presence of any personal, professional, or financial relationships.

## Data Availability

Underlying data are available on Dryad (https://doi.org/10.5061/dryad.vq83bk3ws).
